# Diagnostic Workup of Acute Myeloid Leukemia: What Is Really Necessary? An Italian Survey

**DOI:** 10.3389/fonc.2022.828072

**Published:** 2022-02-17

**Authors:** Maria Teresa Voso, Felicetto Ferrara, Sara Galimberti, Alessandro Rambaldi, Adriano Venditti

**Affiliations:** ^1^ Department of Biomedicine and Prevention, Tor Vergata University, Rome, Italy; ^2^ Division of Hematology, Cardarelli Hospital, Naples, Italy; ^3^ Department of Clinical and Experimental Medicine, Section of Hematology, University of Pisa, Pisa, Italy; ^4^ Department of Oncology-Hematology, University of Milan, Milan, Italy; ^5^ Azienda Socio Sanitaria Territoriale Papa Giovanni XXIII, Bergamo, Italy

**Keywords:** acute myeloid leukemia, molecular genetics, AML diagnostics, genetic subtypes, multidisciplinary diagnostics, morphology, mutational analysis

## Abstract

Acute myeloid leukemia (AML) is a heterogeneous disease with a wide variety of clinical presentations, morphological features, and immunophenotypes. The diagnostic approaches to AML that are adopted in Italy have been explored using an online Delphi-based process to expand the global discussion on mandatory tests for the correct diagnosis and, consequently, for optimal management of AML in clinical practice. The final results of the panel of Italian hematologists involved in this work highlight the importance of genetic evaluation for classification and risk stratification and firmly establish that karyotyping, fluorescence *in situ* hybridization in cases with non-evaluable karyotype, and molecular tests must be performed in every case of AML, regardless of age. Obtaining clinically relevant genetic data at diagnosis is the basis for the success of patient-tailored therapy. The Italian specialists also confirm the role of multidisciplinary diagnostics for AML, now mandatory and expected to become more important in the future context of “precision” medicine.

## Introduction

Acute myeloid leukemia (AML) is an uncontrolled clonal proliferation derived from progenitor/precursor hematopoietic cells. Abnormal differentiation of myeloid cells results in high levels of immature malignant cells (blasts) and fewer normally differentiated red blood cells, white blood cells and platelets (1, 2). From a genetic point of view, the definition of AML includes heterogeneous entities, with a wide variety of clinical presentations, morphological features, immunophenotypes. Genetic abnormalities, including chromosomal abnormalities and mutations in proliferation/survival mechanisms (*FLT3*) and differentiation/apoptosis pathways (*CEBPA*, *RUNX1*, and *NPM1*), play a pathogenetic role in AML, provide prognostic criteria, and can guide therapy (3–5). In recent years, relevant progress has been made not only in understanding the pathogenesis of the disease but also in the development of diagnostic tools and novel therapies (6).

The most recent World Health Organization (WHO) classification, published in 2016, compared with that published in 2008 (7), included new AML entities with different cytogenetic and molecular characteristics (8). Moreover, the prognostic and diagnostic relevance of the new molecular features were reviewed and integrated into existing sets of criteria. Six categories of AML were identified: (1) AML with recurrent genetic abnormalities, (2) AML with myelodysplasia-related changes (AML-MRC), (3) therapy-related myeloid neoplasms, (4) AML not otherwise specified (AML-NOS), (5) myeloid sarcoma, and (6) myeloid proliferations related to Down syndrome (8).

Several organizations, including the National Comprehensive Cancer Network (NCCN), the European Leukemia Net (ELN), and the European Society for Medical Oncology (ESMO), recently developed guidelines and risk score systems to help physicians to design ab initio the best “patient-tailored” therapeutic strategy (9–11). Interestingly, all these risk-stratification systems and guidelines are based on chromosomal abnormalities and gene mutations.

The diagnostic process of a heterogeneous disease such as AML is complex and requires high levels of expertise. A panel of Italian hematologists explored the diagnostic approaches that are adopted across the national territory through a survey that involved a representative sample of the Italian Centers of Hematology.

Information on how Italian hematologists manage the diagnostic workup of AML may contribute to expand the global discussion on mandatory tests for the correct diagnostic approach and, consequently, for optimal management of AML in clinical practice. The diagnosis of the different genetic subtypes of AML is a subject of intense debate within the international scientific community because it has relevant prognostic and therapeutic implications.

## Methods

To gather expert opinion on the implementation of accurate diagnosis of AML subtypes, we used an online Delphi-based process (Estimate-Talk-Estimate). This is a group-facilitative method designed to verify the convergence of opinion of a panel of experts in a given area of uncertainty within health-related research (12, 13).

The process was developed over nearly 7 months using the following steps: (1) establishment of a scientific steering committee of five experts who were in charge of reviewing the literature and developing the survey items; (2) selection of a panel of AML specialists; (3) administration of online survey to the panel of AML specialists; (4) collection and analysis of the results; (5) final meeting.

### Scientific Steering Committee

Five experts from Italian hematology institutions were identified as representatives of the specialists involved in the medical care of patients with AML. The scientific steering committee defined 26 questions covering four main topics:

Topic 1: application of the WHO 2016 classification (8 items)

Topic 2: confirmatory diagnostic tests (4 items)

Topic 3: implementation of the guidelines (7 items)

Topic 4: therapeutic implications (7 items)

The possible answers to each question were chosen from multiple options, all possible and applicable in clinical practice.

### Panel of AML Specialists

Forty-eight specialists (hematologists, cytogeneticists, and molecular biologists) selected from 42 centers among the Italian hematology institutions, were invited to participate in the project during a virtual meeting where the project objectives and methodology were shared.

### Administration of the Online Survey

The survey elaborated by the steering committee was delivered to the panel of AML specialists: 34 participants from 31 sites (29 hematologists and 5 cytogeneticists/molecular biologists) expressed an opinion on each of the 26 questions, independently and blindly. All 34 specialists were affiliated to centers with availability of a diagnostic lab, and with an allogeneic or autologous stem cell transplantation unit in 27 and 7 cases, respectively. The survey was performed online on a secure website using an online platform.

### Final Meeting

The scientific steering committee collected and analyzed the results, then discussed the real-life AML diagnostic approach adopted in Italy in the light of the international recommendations.

## Results

The panel of specialists answered each of the 26 questions based on their own clinical practice, experience, and facilities available (**Table 1**).

**Table 1:** List of the 26 questions included in the survey for the four main topics.

Topic 1: Application of the WHO 2016 Classification	
1.	In daily clinical practice, do you use the WHO 2016 classification when diagnosing AML?
2.	In light of the 2016 WHO classification, which tests do you perform to diagnose AML?
3.	When collecting anamnestic information in a patient with a newly diagnosed AML, what information do you require in detail?
4.	For the diagnosis of “AML with recurrent genetic abnormalities,” the WHO 2016 classification requires cytogenetic and molecular analysis. While waiting for the cytogenetic report, what is your approach regarding the detection of genetic anomalies with molecular techniques?
5.	Among the “AML with recurrent genetic abnormalities,” the WHO 2016 classification includes AML with the biallelic CEBPA mutation. How do you identify this mutation in your laboratory?
6.	Among the “AML with recurrent genetic abnormalities,” the WHO 2016 classification includes AML with the biallelic CEBPA mutation. Your opinion about flow cytometry is that:
7.	The 2016 WHO classification also includes “AML with MDS-related abnormalities (AML-MRC)”. Do you think it is possible to diagnose these AML subtypes with microscopic observation only?
8.	The 2016 WHO classification also includes “AML with MDS-related abnormalities (AML-MRC)”. In case the karyotype is not available (punctio sicca) or it takes several days to report, do you think that FISH could be a valid alternative?
	**Topic 2: confirmatory diagnostic tests**
9.	Do you think that in AML categories genetically identified by specific abnormalities, the definition of the marrow blast count is still necessary?
10.	In your center, what is the average cytogenetic reporting time?
11.	In your center, what is the average molecular reporting time for PML-RARa?
12.	In your center, what is the average reporting time for the most common genetic abnormalities (BCR-ABL1, FLT3, NPM1, RUNX1-RUNX1T1, CBFB-MYH11)?
**Topic 3: Implementation of the Guidelines**	
13.	According to the 2017 ELN guidelines, which analyses do you perform for prognostic stratification?
14.	The 2017 ELN guidelines use the mutations of RUNX1, ASXL1, TP53 to classify a patient as a “high risk” subject. Do you perform tests to identify these mutations?
15.	According to the ESMO 2020 guidelines, test of FLT3 mutations must be quickly performed. How long does it take to get the report in your center?
16.	According to the ESMO 2020 guidelines, test of FLT3 mutation must be quickly performed. What is the most frequently used methodology in your center?
17.	In AML with NPM1 mutation, if the FLT3-ITD mutation is also present, how do you stratify the patient’s risk?
18.	The ESMO 2020 guidelines also recommended performing tests for IDH1 and IDH2 mutations. What is the methodology in use in your laboratory?
19.	The ESMO 2020 guidelines also recommended performing tests of IDH1 and IDH2 mutations. Do you think it is useful to perform it in the routine clinical practice?
**Topic 4: Therapeutic Implications**	
20.	In your daily clinical practice, which of the following guidelines do you use for AML treatment?
21.	Do you think that the definition of an AML as AML-CBF has a significant impact on the therapeutic strategy?
22.	Do you think that the definition of an AML as AML-MRC has a significant impact on the therapeutic strategy?
23.	In a patient eligible for intensive chemotherapy with FLT3-mutated AML-MRC, which therapeutic choice do you usually prefer?
24.	In an AML FLT3 wild-type at diagnosis, do you think it is mandatory to perform the FLT3 research at relapse?
25.	Could the assessment of minimal residual disease have an impact on the AML therapeutic management?
26.	Which methodology do you use to evaluate minimal residual disease, in order to manage AML treatment more appropriately?

### Topic 1: Application of the WHO 2016 Classification

In recent decades, the diagnostic workup for AML, initially based solely on morphological examination, has integrated more and more disciplines and methodologies. The WHO 2016 classification adopted such fundamental tools as cytomorphology, cytochemistry, immunophenotyping, cytogenetics, and molecular genetics. The integration of all these techniques allows for a comprehensive and complementary characterization of each patient with AML, which is a prerequisite for optimal diagnosis and management.

The aim of the questions in the first topic of the Delphi questionnaire was to investigate the specialists’ attitude in applying the WHO diagnostic criteria in clinical practice, focusing on which tests they would consider crucial for the correct identification of AML subtypes (**Table 1**).

### Panelists’ View and Behavior in AML Clinical Practice

Almost all experts participating in the survey stated that they always refer to the WHO 2016 classification (**Table 2**) for the diagnosis of AML. Generally, despite differences in terms of technological equipment among the centers, there was stringent observance of the WHO 2016 recommendations. However, some differences also emerged; for example, 26% of the specialists perform morphology, immunophenotyping, conventional cytogenetics, and mutational analysis of *NPM1*, *CEBPA*, and *RUNX1* genes only in young patients. However, the specialists who provided comments in the open field stated that the use of tests is driven more by fitness to undergo intensive chemotherapy than by age. On the other hand, 35% of the specialists perform all of the above diagnostic tests, regardless of age. Finally, 39% of specialists stated that assessment for *CEBPA* and *RUNX1* is performed in selected cases only.

**Table 2:** The WHO 2016 classification of acute myeloid leukemia (AML).

AML with recurrent genetic abnormalities	AML with t(8;21)(q22;q22.1);*RUNX1-RUNX1T1*
	AML with inv(16)(p13.1q22) or t(16;16)(p13.1;q22);*CBFB-MYH11*
	APL with *PML-RARA*
	AML with t(9;11)(p21.3;q23.3);*MLLT3-KMT2A*
	AML with t(6;9)(p23;q34.1);*DEK-NUP214*
	AML with inv(3)(q21.3q26.2) or t(3;3)(q21.3;q26.2);*GATA2*,*MECOM*
	AML (megakaryoblastic) with t(1;22)(p13.3;q13.3);*RBM15-MKL1*
	*Provisional entity: AML with BCR-ABL1*
	AML with mutated *NPM1*
	AML with biallelic mutations of *CEBPA*
	*Provisional entity: AML with mutated RUNX1*
AML with myelodysplasia-related changes	
Therapy-related myeloid neoplasms	
AML, not otherwise specified	AML with minimal differentiation
	AML without maturation
	AML with maturation
	Acute myelomonocytic leukemia
	Acute monoblastic/monocytic leukemia
	Pure erythroid leukemia
	Acute megakaryoblastic leukemia
	Acute basophilic leukemia
	Acute panmyelosis with myelofibrosis
Myeloid sarcoma	
Myeloid proliferations related to Down syndrome	

When collecting anamnestic information, most of the specialists (81%) declared that they carefully seek information about previous exposure to chemo-/radiotherapy to exclude therapy-related myeloid neoplasms. An accurate anamnesis, also asking about history of a previous transfusion support or a diagnosis of antecedent myelodysplastic syndrome (MDS), was considered a fundamental step for the identification of AML-MRC.

The turnaround time to obtain information on cytogenetics can be as long as 7–15 days, whereas it can be no longer than 72 h for molecular testing. The specialists were asked to state which molecular changes they considered critical for the diagnosis of AML with recurrent genetic abnormalities. There was no convergence on which molecular assay to request. About one third (36%) of the panelists claimed to request only qualitative polymerase chain reaction (PCR) for the main fusion genes (*RUNX1-RUNX1T1*, *CBFB-MYH11*, *PML-RARalpha*, *BCR-ABL1*). The remaining two thirds were equally divided between those who request all possible PCR assays (32%) and those who urgently require only qualitative PCR for *PML-RARalpha* (32%) to exclude a diagnosis of acute promyelocytic leukemia.

**Figure 1:**
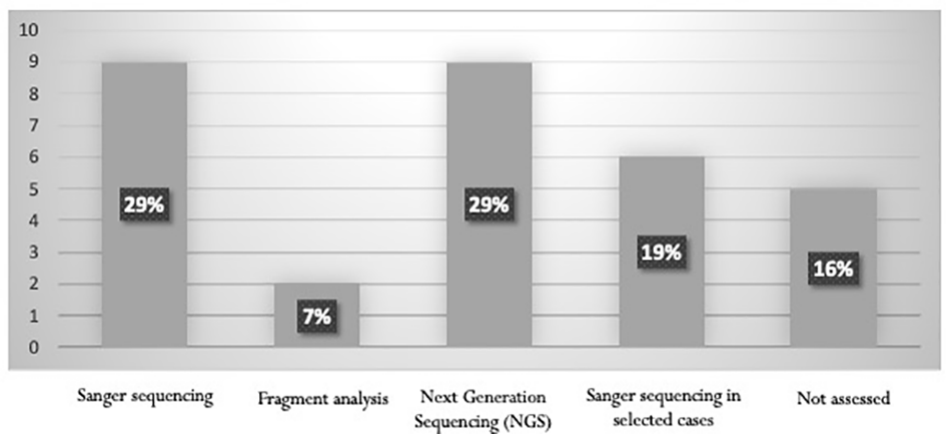
Methods used for the detection of CEBPA mutation status.

Sanger sequencing (29%) and next-generation sequencing (NGS) (29%) are the most frequently used techniques for detecting AML with biallelic *CEBPA* mutation (**Figure 1**). According to the panelists, the execution of the molecular assays is mandatory. Sixty-five percent the panelists stated that the use of flow cytometry, even with CD7 assessment, is not reliable enough for the identification of biallelic *CEBPA* mutations (14).

Over 70% of panelists believed that morphological assessment alone is not sufficient to support the diagnosis of AML-MRC. Morphology assessment should always be associated with clinical history, cytogenetics *NPM1* and biallelic *CEBPA* mutational analysis, in line with WHO recommendations. Sixty percent of the specialists mentioned fluorescence *in situ* hybridization (FISH) as a valid substitute for karyotyping to clarify difficult diagnosis such as AML-MRC as soon as possible. Such an approach is deemed reliable in situations of *punctio sicca*, insufficient number of metaphases, or when the turnaround time to obtain information about the karyotyping is too long.

### Comments From the Scientific Steering Committee

The outcomes from the first set of answers demonstrated the specialists’ attitude to applying the 2016 WHO AML classification and the recommended diagnostic workup. Nevertheless, such an intention is affected by the technological heterogeneity among the different centers and the use or not of reference laboratories (such as GIMEMA LabNet AML, https://www.gimema.it/ricerca/labnet/) for centralized activities.

It is also evident that the prognostic/therapeutic implications are strong drivers for the specialists to decide which molecular changes should be tested. It may be important to reinforce the existing differences between the WHO 2016 classification, which defines specific AML subtypes by focusing on cytogenetic and molecular features, and the ELN scoring system, which uses the same disease-specific biological aspects to distinguish patients with a “favorable”, “intermediate” or “adverse” risk (6, 8) prognosis.

Several areas of debate emerged from the survey. Medical history appears to be a fundamental starting point in patients’ assessment. In addition to previous hematologic diseases (even undiagnosed) and cytotoxic treatments, there is increasing evidence of a frequent association between bone marrow dysplastic features and autoimmune disorders (15). Therefore, it may be important to consider this aspect in the patient’s history as immediately suspicious for AML-MRC.

With regard to age, the participants stated that it is not an absolute discriminating factor. The impact of age should be evaluated in the light of co-existing co-morbidities, with the aim of selecting patients eligible for intensive chemotherapy and, if needed, allogeneic transplantation. Nevertheless, most new targeted agents are also indicated for the treatment of older patients with AML (16–18), therefore accurate molecular characterization of the disease is desirable regardless of age (19).

With regard to the diagnosis of AML with recurrent genetic abnormalities, molecular assays covering the spectrum of the most relevant gene rearrangements are valuable tools to assist the diagnostic process while waiting for the cytogenetic results. Furthermore, some gene rearrangements such as *RUNX1-RUNX1T1*, *CBFB-MYH11*, *PML-RARalpha*, *BCR-ABL1* are also clinically relevant because they serve to track measurable residual disease (MRD) during and after treatment (20–22).

For AML with recurrent genetic abnormalities, the WHO 2016 classification includes cases with biallelic *CEBPA* mutations. There is a need to identify these mutations because they could have a diagnostic or prognostic role (8, 23, 24). From a diagnostic point of view, the presence of mutations in the *CEBPA* gene should make clinicians consider a condition of inherited predisposition to AML and prompt screening of family members. Such information is also crucial for the choice of donor in cases of hematopoietic stem cell transplantation (25). From a prognostic standpoint, it is necessary to establish the mutational pattern of *CEBPA* in order to adopt an appropriate post-induction strategy (26, 27). *CEBPA* mutation confers a favorable prognosis if present in the biallelic pattern.

Morphology remains a fundamental diagnostic step and contributes to the identification of subtypes of AML. Nevertheless, classification of AML-MRC depends on a multidisciplinary diagnostic approach. In addition to morphological assessment, necessary for evaluating the presence of bone marrow dysplastic features, a correct diagnosis of AML-MRC requires cytogenetics to identify cases of *de novo* AML with MDS-related cytogenetic abnormalities (**Table 3**), and exclude recurrent cytogenetic abnormalities (**Table 2**), anamnestic investigation, and molecular analysis to exclude *NPM1* and biallelic *CEBPA* mutations (8, 28).

**Table 3:** Cytogenetic abnormalities for the diagnosis of AML with myelodysplasia-related changes.

Complex karyotype	3 or more abnormalities
Unbalanced abnormalities	-7/del(7q)
	Del(5q/t(5q)
	i(17q)/t(17p)
	-13/del(13q)
	del(11q)
	del(12p)/t(12p)
	idic(X)(q13)
Balanced abnormalities	t(11;16)(q23;p13.3)
	t(3;21)(q26.2;q22.1)
	t(1;3)(p36.3;q21.2)
	t(2;11)(p21;q23.3)
	t(5;12)(q32;p13.2)
	t(5;7)(q32;q11.2)
	t(5;17)(q32;p13.2)
	t(5;10)(q32;q21.2)
	t(3;5)(q25.3;q35.1)

Even though the specialists agree that conventional karyotyping is the reference technique and every effort should be made to obtain its results, they often use FISH analysis in cases of unknown karyotype or to bypass a long turnaround time. FISH provides a targeted and fast approach to obtain diagnostic and prognostic information, especially regarding chromosomes 3, 5 and 7 (29).

### Topic 2: Confirmatory Diagnostic Tests

The intrinsic complexity of the diagnostic workup of AML entails some critical issues, such as the availability of adequate technical resources and facilities and the turnaround time to obtain the results. In addition to affecting the diagnostic process, these factors have a relevant impact on the therapeutic decisions. Questions asked in the second topic of the survey focused on the time necessary to complete a full laboratory assessment and on the identification of potential solutions (**Table 1**).

### Panelists’ View and Behavior in AML Clinical Practice

Sixty-five percent of the panelists agreed that, in categories identified by specific genetic abnormalities, the assessment of bone marrow blast count is still relevant, whereas 35% of the AML specialists considered cytomorphology, in the presence of specific genetic lesions, not mandatory or vicariable by flow cytometry.

**Figure 2:**
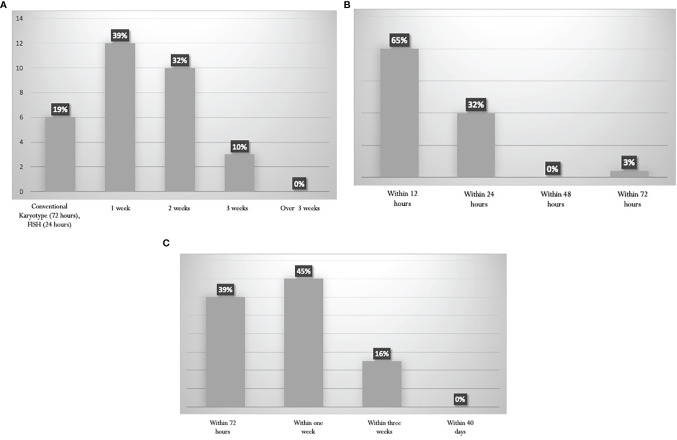
Turnaround times for the diagnostics procedures. **(A)** Karyotype. **(B)** PML-RARalpha rearrangement by RT-PCR. **(C)** Other Recurrent genetic abnormalities (BCR-ABL1, FLT3, NPM1, RUNX1-RUNX1T1, CBFB-MYH11).

The turnaround time for the cytogenetic reports was variable. Results are shown in **Figure 2A**. Information about *PML-RARalpha* is achieved within 24 hours in 97% of the Italian laboratories (**Figure 2B**). The turnaround time for the other common genetic abnormalities (*BCR-ABL1*, *FLT3*, *NPM1*, *RUNX1-RUNX1T1*, *CBFB-MYH11*) is highly variable (**Figure 2C**), ranging from 72 hours (39% of the panelists) to 1 week for non-NGS reports (45% of the panelists), up to 3 weeks for NGS reports (16%).

### Comments From the Scientific Steering Committee

The scientific steering committee emphasized the importance of bone marrow (BM) blast count. In patients with specific genetic alterations, cytomorphological examination of BM, and accurate evaluation of blast infiltration, according to the current guidelines, is of paramount importance to guide diagnosis and assess response to treatment (6). The committee also emphasizes that in the case of t(15;17), t(8;21), inv(16), or t(16;16) and their respective fusion genes, the diagnosis of AML can be made even if the BM blast percentage is less than 20%.

The ELN recommends that the turnaround time for reporting *NPM1* and *FLT3* mutation should be 48–72 hours, 96 hours for further gene rearrangements, 24 hours for molecular reporting of *PML-RARalpha*, and 5–7 days for cytogenetics (11). These turnaround times are generally met by most centers (**Figure 2**).

The committee believes that there is still room for improvement and a strategy to reduce variability among sites should consider the use of laboratory networks, such as the GIMEMA LabNet AML network, to assist in the diagnostic process of AML by centers not fully equipped for complex molecular tests. Finally, the committee agree that genetic assessment for AML should be repeated in cases of disease relapse to demonstrate clonal evolution of the disease, including acquisition of chromosomal abnormalities, the disappearance or appearance of somatic mutations, including *FLT3* mutations, where the emergence of mutated clones paves the way for the delivery of new *FLT3* inhibitors given as single agents, as gilteritinib.

### Topic 3: Implementation of the Guidelines

The questions for the third topic explored how the specialists apply cytogenetic and molecular analyses to risk-stratify cases of AML (**Table 1**) and to what extent they rely on current recommendations.

### Panelists’ View and Behavior in AML Clinical Practice

**Figure 3:**
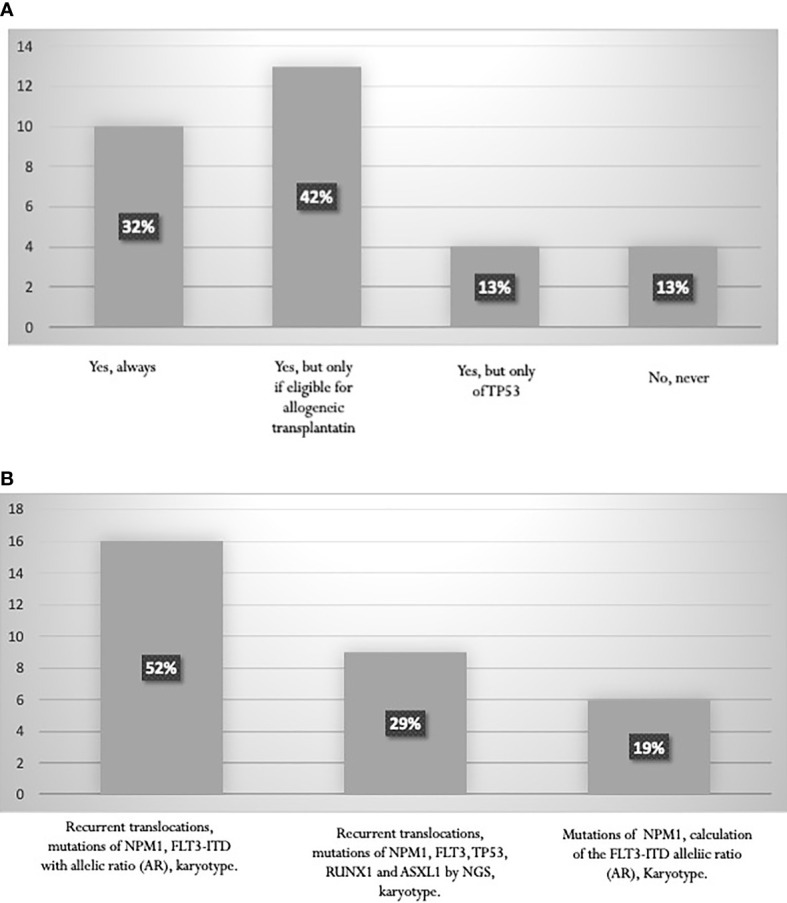
**(A)** Proportion of laboratories performing RUNX1, ASXL1, TP53 mutation studies. **(B)** Tests performed for AML stratification according to ELN 2017.

The search for recurrent translocations with rapid techniques, karyotype assessment, study of the mutational state of *NPM1* and calculation of the *FLT3-ITD* allelic ratio (AR) are fundamental for prognostic stratification. According to the 2017 ELN guidelines (6), a high percentage (74%) of AML experts perform molecular analyses of *RUNX1*, *ASXL1*, *TP53* genes for a proper definition of the “adverse risk” category. This percentage includes 32% of experts who always perform these analyses and 42% who carry out them specifically to establish eligibility for allogeneic transplantation (**Figure 3A**). Only 29% of the specialists stated that NGS data are available at diagnosis, and can classify patients within ELN categories before starting treatment (**Figure 3B**). On the contrary, mutation status by NGS becomes available in most cases at the end of induction, and is used to choose post-consolidation strategies, including allogeneic stem cell transplantation.

**Figure 4:**
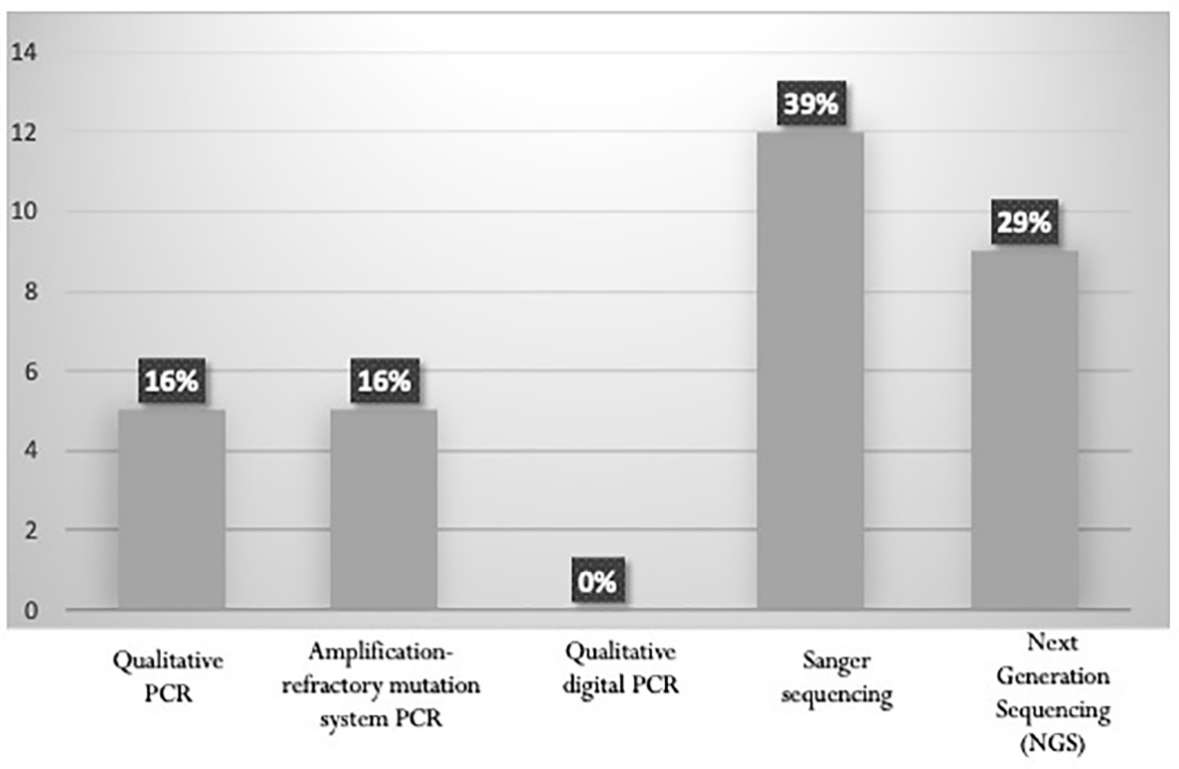
Proportion of Laboratories assessing IDH1/IDH2 mutation status according to ESMO 2020 guidelines.

Almost all panelists comply with the indications of the ESMO 2020 guidelines and perform analysis for *FLT3* mutations using fragment analysis for *FLT3-ITD*, and digestion with the ECO-RV restriction enzyme for FLT3-TKD. In *NPM1-*mutated AML, 71% of the panelists apply the ELN recommendations, indicating that the presence of *FLT3-ITD* mutation with an AR <0.5 is a favorable prognostic factor. The ESMO 2020 guidelines also recommends testing *IDH1* and *IDH2* mutations. Across the laboratories involved in this survey, the assay is mainly performed by Sanger sequencing or NGS. The amplification-refractory mutation system PCR is used in 16% of the centers (**Figure 4**).

### Comments From the Scientific Steering Committee

The widespread use of NGS, although not homogeneously distributed across the national territory, has a relevant impact on the laboratory activities of the centers, because this method entails considerable complexity with report accuracy and data interpretation. The implementation of NGS in clinical practice will certainly contribute to expand the body of information necessary for a proper application of the ELN risk stratification. However, cytogenetics and molecular biology testing should not be omitted; rather, they should be complemented by NGS.

The committee agree that assessment of *IDH1* and *IDH2* mutation, although useful for a comprehensive genetic characterization of AML (11), is not urgent at the time of diagnosis. This data does not have a therapeutic impact due to the current unavailability of IDH1/IDH2 inhibitors in Europe. Therefore, even though guidelines present decisive drivers for diagnosis, prognosis, and treatment management, they should be adapted to the national clinical practice and the real likelihood that the patients can have access to new agents, based on the National Health System regulations in force. At present, IDH mutational analysis can however help physicians to select for specific treatment combinations, in particular including hypomethylating agents and venetoclax, since these mutations have been associated with improved response and survival (30).

### Topic 4: Therapeutic Implications

The last section of the survey focused on the impact that international guidelines and identification of AML subtypes might have on the therapeutic strategy. Considering that, in recent years, robust evidence of the prognostic role of MRD has emerged, specific questions on this issue were asked to understand to what extent MRD-driven therapeutic strategies are adopted among the centers (**Table 1**).

### Panelists’ View and Behavior in AML Clinical Practice

Sixty-one percent of the specialists involved in the Delphi project rely on internal guidelines for AML management (PDTA, diagnosis, therapy, and assistance pathway), and 36% and 3% stated that they refer to the ESMO (11) and NCCN guidelines (9), respectively.

The specialists almost unanimously confirmed that the identification of disease genetic subtypes, such as those characterized by the rearrangement of core binding factors (AML-CBF), APL and AML-MRC, has a significant impact on clinical decisions. For patients considered eligible for intensive chemotherapy, with a diagnosis of AML-MRC and concomitant *FLT3* mutation, 80% of the clinicians opted for targeted induction treatment (“7+3” regimen plus midostaurin) (31) rather than an AML-MRC-specific treatment such as CPX-351 (32).

The panelists unanimously stated that *FLT3* re-testing is critical upon relapse to offer FLT3 inhibitor treatment if the mutation occurs (33, 34). In addition, all the experts agreed that MRD assessment should be mandatory in clinical practice because it could affect the therapeutic management of AML. Seventy-four percent of the respondents believed that the technique to use for MRD assessment depends on the presence of a molecular marker; 16% of the specialists believed that flow cytometry still has a role, especially in selected situations, and 10% stated that they prefer to use molecular analysis when applicable (35).

The experts agreed on the potential predictive role of hyper-expression of Wilms tumor gene (*WT1*) (36), even if it is not included in the lists of genes that can be adopted for MRD assessment.

### Comments From the Scientific Steering Committee

As observed across the survey, the 2016 WHO classification and identification of AML genetic subtypes seem to play a fundamental role in therapeutic decisions in all centers. Nevertheless, the choice of a therapy for a specific patient with AML who would be eligible for multiple treatments is still a complex process. On the one hand, the presence of a specific mutation or a specific immunophenotype, may indicate the use of targeted agents, as f.i. TKi or Mylotarg in CD33+ AML. On the other hand, the complex genetic background should be also considered, especially in the light of the patient’s clinical history, and in AML-MRC. In conclusion, it would be desirable to carry out a complete “biological” characterization of patients in order to choose the most appropriate treatment among those available. For example, for *FLT3*-mutated cases, both midostaurin and CPX-351, despite their diverse mechanisms of action, are effective (32, 37). Midostaurin has been developed specifically for *FLT3-*mutated AML but data concerning MRC- or therapy-related subtypes are still inconclusive (36). CPX-351, indicated for MRC- and therapy-related AML (32), also seems to be effective in *FLT3*-mutated cases, as reported by the French (38) and Italian (39) groups.

With regard to MRD, at diagnosis, AMLs are characterized by genotypical and phenotypical features that allow leukemic cells to be distinguished from their normal counterparts (40). The leukemia-associated immunophenotype “ (LAIP) identified by multi-color flow cytometry or the “different from normal” pattern can then be used to monitor MRD (41, 42). Moreover, in selected cases, such as those negative for recurrent rearrangements or *NPM1* mutations, overexpression of *WT1* can be considered for MRD monitoring (43–48). However, *WT1* is not completely adequate for MRD, because a reproducible and validated cut-off to distinguish between normal and increased values is still not available (49), and, due to low sensitivity and specificity of the test, it is not yet recognized as a valuable method, unless no other MRD markers are available (50). Because of the solid evidence that the presence of MRD after cytoreductive therapy does affect patient outcome, it seems reasonable that it should also influence treatment decisions (51, 52). In this respect, increasing evidence suggests that outcomes may be improved using a MRD-driven therapeutic strategy, also including allogeneic stem cell transplantation for patients persistently MRD positive or re-converted from MRD negative to MRD positive (53).

The prospective GIMEMA AML1310 trial proposed for patients eligible for intensive chemotherapy is a risk- and MRD-adapted therapeutic project, integrating the upfront genetic features and the post-consolidation MRD (54). Patients at “favorable risk” (those included in the favorable ELN risk group and those at intermediate risk or MRD-negative at consolidation) received autologous stem cell transplantation, whereas patients at “poor risk” (ELN poor risk and intermediate risk but still MRD-positive at consolidation) underwent allogeneic transplantation. As expected, 2-year overall survival and disease-free survival were significantly different between ELN favorable and poor risk categories (overall survival 74% vs 42%, disease-free survival 61% vs 45%). Concerning the “intermediate” cohort, the ELN risk- and MRD-adapted strategy improved outcomes to rates observed in the “favorable” category (overall survival, 79% for MRD negative and 70% for MRD-positive cases; disease-free survival, 61% and 67%, respectively), thus confirming a role for autologous transplantation in the “favorable” category and for allogeneic transplant in cases at novel “poor” risk (55).

## Conclusions

The diagnostic workup of AML resulting from this Delphi project (which mirrors Italian hematologic centers in practice) is the product of an integrated approach, primarily based on international guidelines, including patient history, and morphological, immunophenotyping, and cytogenetics/molecular findings. The opinions of the panelists and experts suggest that only a holistic approach to patients with AML might lead to long-lasting therapeutic success.

The originality of this work lies in the initial choice of Italian experts to develop a project aimed at the definition of the best diagnostic process for patients with AML subtypes starting from the assumption that a change of paradigm is needed by some centers that deal with AML. In particular, awareness is increasing to ensure that complete and “performant” diagnostics are fundamental from the first steps of the decision-making process. Morphologic evaluation of bone marrow and peripheral blood smears still plays a decisive diagnostic role in AML. The importance of genetic evaluation, both for classification and for risk stratification, firmly establishes that karyotyping, FISH, and molecular tests have to be performed in every case of AML, independently of age. Furthermore, genetics is continuously evolving and new information becomes available in the scientific community. It has recently been shown that some cases of clinically defined *de novo* AML are characterized by a chromatin-spliceosome mutational signature and, from a clinical and prognostic perspective, resemble a secondary more than *a de novo* entity, despite the absence of specific morphological and cytogenetic abnormalities (54). The updated knowledge of the genetic aspects of AML will determine further improvements in the WHO classification, with the possible inclusion of new molecular entities.

Another important aspect is represented by the interdisciplinary approach and the opportunity to create an efficient network between different players in the AML diagnosis (hematologist, cytogeneticist, pathologist, molecular biologist), to harmonize reports, increase the quality of technologies, and share knowledge. We emphasize the role of multidisciplinary diagnostics in AML, which is mandatory today and will gain even more importance in the future, especially in the context of precision medicine. This approach, together with the identification of centralized laboratories that could help centers not technically fully equipped, will overcome the possible problem of turnaround times, which sometimes vary from some hours to a week in different laboratories. Recent data confirm that it may be a feasible strategy to wait for genetic and other laboratory test results in clinically stable patients to assign them to the best available treatment option (56). Therefore, the possibility of timely informative and clinically relevant genetic and diagnostic results at diagnosis represents the basis for the success of the patient-tailored therapy, made possible today by the introduction of many new targeted therapies in the clinical armamentarium.

Demostrating that the diagnostic approaches in AML are not uniform across the Italian territory, this survey highlights the fundamental role of the GIMEMA LabNet and, in general, of a diffuse lab network to support the clinical Centers which are not technically fully equipped, and to finally ensure an adequate management of AML.

## Data Availability Statement

The original contributions presented in the study are included in the article/supplementary material. Further inquiries can be directed to the corresponding author.

## Author Contributions

MV designed the project, selected the literature, wrote and reviewed the manuscript. FF designed the project, selected the literature, wrote and reviewed the manuscript. SG designed the project, selected the literature, wrote and reviewed the manuscript. AR designed the project, selected the literature, wrote and reviewed the manuscript. AV designed the project, selected the literature, wrote and reviewed the manuscript. All authors contributed to the article and approved the submitted version.

## Conflict of Interest

AR has received travel expenses and fees for consultancies and participation in meetings, boards and symposia sponsored by Jazz, ABBVIE, Amgen, Pfizer, Celgene, Novartis, Incyte, Omeros, Astellas, and Roche. AV has served on an advisory board, been an invited speaker, and taken part in a speakers’ bureau for Novartis, Jazz, Pfizer, Astellas, Gilead, Helsinn, and Amgen. SG has received speaker fees for events supported by Amgen, Jazz, Astellas, and Novartis. MV has served on an advisory board, been an invited speaker, and taken part in a speakers’ bureau for Novartis, Jazz, Astellas, BMS/Celgene.

The remaining author declares that the research was conducted in the absence of any commercial or financial relationships that could be construed as a potential conflict of interest.

## Publisher’s Note

All claims expressed in this article are solely those of the authors and do not necessarily represent those of their affiliated organizations, or those of the publisher, the editors and the reviewers. Any product that may be evaluated in this article, or claim that may be made by its manufacturer, is not guaranteed or endorsed by the publisher.
